# Whole-Genome Sequencing of Pig Gut Lactobacillus amylovorus CICC 6090

**DOI:** 10.1128/mra.01197-22

**Published:** 2023-03-20

**Authors:** Yeshi Yin, Zongyan Li, Huahai Chen

**Affiliations:** a Key Laboratory of Comprehensive Utilization of Advantage Plants Resources in Hunan South, Hunan Engineering Research Center for Research and Development of Plant Resources in Nanling Area, College of Chemistry and Bioengineering, Hunan University of Science and Engineering, Yongzhou, Hunan, China; b School of Biological Science and Technology, Hunan Agricultural University, Changsha, Hunan, China; University of Rochester School of Medicine and Dentistry

## Abstract

Lactobacillus amylovorus has been reported to reduce weight and fat content in humans. To identify and understand the mechanism underlying this process, the complete genome of CICC 6090, which was isolated from pig intestines, was sequenced using PromethION and next-generation sequencing.

## ANNOUNCEMENT

Lactobacillus amylovorus is a probiotic candidate organism. Nakamura et al. (1) and Park et al. (2) reported that *L. amylovorus* CP1563 and KU4 can ameliorate diet-induced obesity. Here, we report the complete genomic sequence of *L. amylovorus* CICC 6090 as ordered from the China Center of Industrial Culture Collection. This may improve our understanding of the molecular mechanism underlying its antiobesity effect.

For genome sequencing, freeze-dried CICC 6090 was cultured for 48 h using De Man-Rogosa-Sharpe (MRS) broth under anaerobic conditions at 37°C. The steps in the Oxford Nanopore Technologies (ONT) sequencing workflow were as follows. DNA was extracted with a QIAgene bacterial genome extraction kit; DNA quality was assessed by electrophoresis, Nanodrop spectrophotometers, and a Qubit fluorometer. The 20-kb DNA fragments were selected using Blue Pippin (Sage Science). Adapter ligation was performed using NBD103 and NBD114 kits (ONT). The DNA library was quantified using Qubit. Real-time single-molecule sequencing was performed on a Nanopore PromethION sequencer (R9.4.1). In summary, 131,900 reads (read mean length of 8,421.43 bp, read *N*_50_ of 13,352 bp) and 1,110,786,305 bp of raw data were generated. Guppy (version 5.0.16) was used for ONT base calling, and a mean_qscore_template of ≥7 and sequence length of ≥1,000 bp were used for quality control. In all, 981,310,309 bp of data passed quality control. Flye version 2.9 (https://github.com/fenderglass/Flye; parameter: off) was used for genome assembly (3). Considering the limitations of genomes assembled solely from ONT data, Pilon version 1.23 (https://github.com/broadinstitute/pilon; parameter: off) was used for correction with next-generation sequencing data (4). For next-generation sequencing, MGIEasy universal DNA library prep kit V1.0 (catalog no. 1000005250; MGI) and MGIEasy DNA Clean beads (catalog no. 1000005279; MGI) were used in accordance with the standard protocol. The fragments had an average size of 200 to 400 bp and were end repaired and ligated using adapters. High-quality clean reads of the DNBSEQ-T7RS platform were obtained using fastp (version 0.12.6). A read length of 150-bp, paired end, was selected for error correction, and 1,220,051,400 bp of clean reads was generated. After the removal of redundant reads, Circlator version 1.5.5 (https://github.com/sanger-pathogens/circlator; parameter: fixstart) was used to move the original protein DnaA sequence to the start site of the genome replication to obtain the final genome sequence (5). Default parameters were used for all software except where otherwise specified.

Final findings included one genome contig (1,977,003 bp, GC content of 38.11%) and no plasmids. The average nucleotide identity (ANI) based on BLAST+ was analyzed online using the system default parameters by comparison with the other five *L. amylovorus* genome sequences that had been deposited in GenomesDB (https://jspecies.ribohost.com/jspeciesws/) (6). The ANI with *L. amylovorus* DSM 20531, *L. amylovorus* GRL 1112, *L. amylovorus* 30SC, *L. amylovorus* DSM 16698, and *L. amylovorus* GRL1118 was 97.65%, 96.87%, 96.79%, 96.70%, and 96.65%, respectively. Coding genes (CDS) were predicted using Prodigal (https://github.com/hyattpd/Prodigal; parameter: -p None -g 11) with intact CDS (7). Drug resistance genes and virulence factors were annotated using the Comprehensive Antibiotic Research Database (CARD, https://card.mcmaster.ca/) (8) and the Virulence Factor Database (VFDB, http://www.mgc.ac.cn/VFs/) (9). In all, 1,947 CDS were detected, and no drug resistance genes or virulence factors were found.

### Data availability.

The complete genome sequences were deposited in the National Center for Biotechnology Information (NCBI) under the accession number CP110363 (https://www.ncbi.nlm.nih.gov/nuccore/CP110363). The raw sequences obtained from the Nanopore PromethION sequencer were deposited in the Sequence Read Archive under the BioProject no. PRJNA895949 (https://www.ncbi.nlm.nih.gov/bioproject/PRJNA895949). The raw sequences obtained from the DNBSEQ-T7RS platform were deposited in the Sequence Read Archive under the BioProject no. PRJNA898672 (https://www.ncbi.nlm.nih.gov/bioproject/PRJNA898672).

## References

[1] Nakamura F, Ishida Y, Aihara K, Sawada D, Ashida N, Sugawara T, Aoki Y, Takehara I, Takano K, Fujiwara S. 2016. Effect of fragmented Lactobacillus amylovorus CP1563 on lipid metabolism in overweight and mildly obese individuals: a randomized controlled trial. Microb Ecol Health Dis 27:30312. doi:10.3402/mehd.v27.30312. 27221805 PMC4879181

[2] Park SS, Lee YJ, Kang H, Yang G, Hong EJ, Lim JY, Oh S, Kim E. 2019. Lactobacillus amylovorus KU4 ameliorates diet-induced obesity in mice by promoting adipose browning through PPARgamma signaling. Sci Rep 9:20152. doi:10.1038/s41598-019-56817-w. 31882939 PMC6934708

[3] Kolmogorov M, Yuan J, Lin Y, Pevzner PA. 2019. Assembly of long, error-prone reads using repeat graphs. Nat Biotechnol 37:540–546. doi:10.1038/s41587-019-0072-8. 30936562

[4] Walker BJ, Abeel T, Shea T, Priest M, Abouelliel A, Sakthikumar S, Cuomo CA, Zeng Q, Wortman J, Young SK, Earl AM. 2014. Pilon: an integrated tool for comprehensive microbial variant detection and genome assembly improvement. PLoS One 9:e112963. doi:10.1371/journal.pone.0112963. 25409509 PMC4237348

[5] Hunt M, Silva ND, Otto TD, Parkhill J, Keane JA, Harris SR. 2015. Circlator: automated circularization of genome assemblies using long sequencing reads. Genome Biol 16:294. doi:10.1186/s13059-015-0849-0. 26714481 PMC4699355

[6] Richter M, Rossello-Mora R, Oliver GF, Peplies J. 2016. JSpeciesWS: a web server for prokaryotic species circumscription based on pairwise genome comparison. Bioinformatics 32:929–931. doi:10.1093/bioinformatics/btv681. 26576653 PMC5939971

[7] Hyatt D, Chen GL, Locascio PF, Land ML, Larimer FW, Hauser LJ. 2010. Prodigal: prokaryotic gene recognition and translation initiation site identification. BMC Bioinformatics 11:119. doi:10.1186/1471-2105-11-119. 20211023 PMC2848648

[8] Alcock BP, Huynh W, Chalil R, Smith KW, Raphenya AR, Wlodarski MA, Edalatmand A, Petkau A, Syed SA, Tsang KK, Baker S, Dave M, McCarthy MC, Mukiri KM, Nasir JA, Golbon B, Imtiaz H, Jiang X, Kaur K, Kwong M, Liang ZC, Niu KC, Shan P, Yang J, Gray KL, Hoad GR, Jia B, Bhando T, Carfrae LA, Farha MA, French S, Gordzevich R, Rachwalski K, Tu MM, Bordeleau E, Dooley D, Griffiths E, Zubyk HL, Brown ED, Maguire F, Beiko RG, Hsiao W, Brinkman F, Van Domselaar G, McArthur AG. 2023. CARD 2023: expanded curation, support for machine learning, and resistome prediction at the Comprehensive Antibiotic Resistance Database. Nucleic Acids Res 51 (D1):D690–D699. doi:10.1093/nar/gkac920. 36263822 PMC9825576

[9] Liu B, Zheng D, Zhou S, Chen L, Yang J. 2022. VFDB 2022: a general classification scheme for bacterial virulence factors. Nucleic Acids Res 50:D912–D917. doi:10.1093/nar/gkab1107. 34850947 PMC8728188

